# Interfacial engineered iron oxide nanoring for T2-weighted magnetic resonance imaging-guided magnetothermal-chemotherapy

**DOI:** 10.3389/fbioe.2022.1005719

**Published:** 2022-10-06

**Authors:** Jianfeng Bao, Hui Tu, Jing Li, Yanbo Dong, Le Dang, Korjova Elena Yurievna, Fengshou Zhang, Lei Xu

**Affiliations:** ^1^ School of Medical Technology and Engineering, Henan University of Science and Technology, Luoyang, China; ^2^ Functional Magnetic Resonance and Molecular Imaging Key Laboratory of Henan Province, The First Affiliated Hospital of Zhengzhou University, Zhengzhou University, Zhengzhou, China; ^3^ Office of Science and Technology, Henan University of Science and Technology, Luoyang, China; ^4^ School of Education, Pingdingshan University, Pingdingshan, China; ^5^ Institute of Psychology, The Herzen State Pedagogical University of Russia, Saint Petersburg, Russia; ^6^ Department of Clinical Laboratory, Huai’an Second People’s Hospital, The Affiliated Huai’an Hospital of Xuzhou Medical University, Huai’an, Jiangsu, China

**Keywords:** theranostic nanoparticles, vortex nanoring, magnetic hyperthermia therapy, hyaluronic acid, magnetic resonance imaging

## Abstract

Due to no penetration depth limitation, low cost, and easy control, magnetic nanoparticles mediated magnetic hyperthermia therapy (MHT) has shown great potential in experimental and clinal treatments of various diseases. However, the low heating conversion efficiencies and short circulation times are major drawback for most existing magnetic-thermal materials. Additionally, single MHT treatment always leads to resistance and recurrence. Herein, a highly efficient magnetic-thermal conversion, ferrimagnetic vortex nanoring Fe_3_O_4_ coated with hyaluronic acid (HA) nanoparticles (Fe_3_O_4_@HA, FVNH NPs) was firstly constructed. Additionally, the doxorubicin (DOX) was successfully enclosed inside the FVNH and released remotely for synergetic magnetic–thermal/chemo cancer therapy. Due to the ferrimagnetic vortex-domain state, the ring shape Fe_3_O_4_ displays a high specific absorption rate (SAR) under an external alternating magnetic field (AMF). Additionally, antitumor drug (DOX) can be encapsulated inside the single large hole of FVNH by the hyaluronic acid (HA) shell and quickly released in response the tumor acidic microenvironments and AMF. What’s more, the non-loaded FVNH NPs show good biocompatibility but high cytotoxicity after loading DOX under AMF. Furthermore, the synthesized FVNH can efficiently reduce the transverse relaxation time and enhance negative magnetic resonance imaging (MRI). The impressive *in vivo* systemic therapeutic efficacy of FVNH was also proved in this work. Taken together, the results of this study demonstrate that the synthesized FVNH NPs offer the promise of serving as multifunctional theranostic nanoplatforms for medical imaging-guided tumor therapies.

## Introduction

In recent years, with the vast development of nanotechnology, the biomedical field has been greeted with tremendous excitement, especially for antitumor applications (Mccarroll et al., 2014; Cryer and Thorley, 2019; Barani et al., 2021). Chemotherapy is one of the most common cancer treatments in clinical practice. However, most chemotherapeutics lack the property of tumor targeting, resulting in various side effects (Lindley et al., 1999; Wu et al., 2005). In order to improve the efficiency of the drug and reduce damage to the normal tissues, many researchers have significantly paid diligently (Zhang et al., 2020a; Yang et al., 2020; Cheng et al., 2021). Among various methods employed to overcome this limitation, designing smart nanocarriers, which can load small molecule antitumor drugs, has shown great promise in precise treatments (Vines et al., 2019; Farzin et al., 2020). Thus, carrying material, encapsulating material, and targeting material is essential for intelligent drug transport (Ahn et al., 2018; Yoo et al., 2019). The porous material is one kind of ideal material for drug loading because of the inherent free space, such as hollow SiO_2_ (Zhu et al., 2010; Hu et al., 2015), mesoporous carbon (Hussain and Guo, 2019; Zhang et al., 2020a), liposomes (Al-Jamal and Kostarelos, 2011; Zhao et al., 2019), and metal-organic frameworks (Luo et al., 2019; Lawson et al., 2021; Ni et al., 2022). For the extra encapsulating material, easy to decorated organic polymers, like the polypyrrole, polyethylene glycol, and polydopamine, are commonly used as a gatekeeper to control the drug release (Wen et al., 2017). The implementation of targeted delivery, small molecule ligands, such as folic acid, Arginine-Glycine-Aspartic acid, and specific base sequences (Steichen et al., 2013; Bazak et al., 2015). Thus, the approach of establishing smart drug delivery nanocarriers can be achieved by integrating all the above-mentioned components.

Conventional medical management is two independent operations in cancer diagnosis and therapy (Litwin and Tan, 2017; Waks and Winer, 2019). Thus, usually, two different drugs are needed for different purposes. This may cause some potential problems: the time interval between the diagnosis and treatment process is long, which may miss the best treatment opportunity; additionally, the treatment depends on the initial diagnosis and can not keep pace with the progression of the disease. So it will be much more meaningful for clinical practice if the diagnosis and treatment can be integrated into one activity, which will help the physician to improve the accuracy of the disease treatment and cut the odds of trouble (Kelkar and Reineke, 2011; Lim et al., 2015). Among all approaches presented in the literature, the multifunctional nanomaterials mediated cancer therapy shows great promise (Janib et al., 2010; Zhang et al., 2019). In the past decades, numerous theranostic nanoplatforms have been developed for combing imaging and therapy in one nanocomposite (Madamsetty et al., 2019). The lipid-based nanoparticles, polymeric nanocarriers, and inorganic nanoplatforms are three major classes of basic materials (Zhao et al., 2020). Among them, iron oxide is usually used for multiple purposes due to its biocompatibility and low prices, such as magnetic resonance imaging (MRI) T1 and T2 enhancement, photoacoustic imaging enhancement, near-infrared imaging, and magnetic hyperthermia (MHT), magnetic targeting drug delivery and ferroptosis induced cell death (Semkina et al., 2015; Ge et al., 2016; Zhou et al., 2018). In addition, the iron oxide nanoparticles will show active targeting, intelligent controlled drug release, and extra multimodal imaging after a simple modification.

In this work, we designed and prepared the ferrimagnetic vortex nanoring Fe_3_O_4_@HA nanoparticles (FVNH NPs) enclosing Doxorubicin for T2 MR imaging-guided hyperthermia therapy and chemotherapy. The base nanoring material has a single huge hole at the nanometer size, giving FVNH unique magnetic properties. These include extremely low coercivity and high saturated magnetization, which are prerequisites for high-performance negative MRI imaging and magnetothermal purpose. At the same time, the weak dipole-dipole interactions of the ring structure at the microscopic level behave as a stable colloidal state at the macroscopic level. The properties of synthesized FVNH were fully characterized, and its antitumor therapeutic efficacy was evaluated *in vitro* and *in vivo*. The preliminary findings in this work suggested that DOX-loading FVNH nanocomposite can be used as a novel drug delivery nanocarrier with the potential for cancer theranostic use.

## Materials and methods

### Chemicals and materials

All reagents were of analytical grade and obtained from several different vendors. FeCl_3_, NH_4_SO_4_, NH_4_HPO_4_, polyethylenimine (PEI, molecular weight = 25,000 Da), N-hydroxysuccinimide, and dialysis bag (3,500 Da) were purchased from Aladdin Biochemical Technology Co., Ltd (Shanghai, China), China. The hyaluronic acid (HA, molecular weight = 5,000–10,000 Da) was purchased from Zhanxun Biotechnology Co., Ltd (Xi’an, China). Dimethyl sulfoxide (DMSO) and absolute ethyl alcohol were purchased from Luoyang Chemical Regent Factory (Luoyang, China). 3-(4, 5-dimethylthiazol-2-yl)-2, 5-diphenyltetrazoliun bromide (MTT), doxorubicin hydrochloride (DOX), and dulbecco minimum essential medium (DMEM) medium was purchased from Yuanye Bio-Technology Co., Ltd (Shanghai, China). The calcein acetoxymethyl ester (Calcein-AM) and Propidium Iodide (PI) were obtained from Biyuntian Biotechnology (Shanghai, China). The 4T1 cell line was purchased from the Cell Bank of the Chinese Academy of Sciences. Milli-Q water (18.2 MΩ·cm) was used in all experiments.

### Nanoring α-Fe_2_O_3_ synthesis

The based single porous material, nanoring α-Fe_2_O_3_, was prepared through a hydrothermal approach according to previous studies (Jia et al., 2008; Bao et al., 2022). Briefly, 1 ml 0.5 mol/L FeCl_3_, 500 μl 0.01 mol/L NH_4_HPO_4_, 500 μl 0.03 mol/L NH_4_SO_4_, and 78 ml water were added into the 100 ml Teflon and then stirred severally for 30 min. Then, the Teflon tank was transferred into the high-pressure reactor and heated to 220°C in the silicone oil bath. After the 24-h reaction, the orange product was collected using a centrifugal machine with 10,000°r/min for 10 min. Then the nanoring α-Fe_2_O_3_ was washed three times with water and ethanol, separately, and then dried at 60°C overnight.

### Reduction of nanoring α-Fe_2_O_3_

The nanoring α-Fe_2_O_3_ was reduced into Fe_3_O_4_ by hydrogen. Briefly, the nanoring α-Fe_2_O_3_ was flat out on the horizontal corundum pot and then put the pot in the tube furnace. The reduction reaction was performed with 5% H_2_/Ar mixture gas and kept at 480°C for 2 h. The color of nanoring changed from orange to black, indicating that the hematite has been changed into magnetite and that a magnet can easily collect the final product.

### Coating the nanoring Fe_3_O_4_ with hyaluronic acid and loading doxorubicin

250 mg of previously reduced nanoring Fe_3_O_4_ was dispersed in the de-ionized 100 ml water and then placed in an ultrasonic tank for 30 min at 85°C. Then 50 mg PEI in 10 ml water was added to the above solution, stirring the mixture for another 24 h. After that, the HA was conjugated to the Fe_3_O_4_@PEI surface according to previous work (Li et al., 2014). For coating the HA, 200 mg HA was dissolved in 20 ml of water at a temperature of 70°C. After cooling to room temperature, 20 ml DMSO with 20 mg carbodiimide and 10 mg N-hydroxysuccinimide was added to the HA solution with vigorous magnetic stirring for 3 h. Then activated HA mixture was put into the previous Fe_3_O_4_@PEI solution using the drop-by-drop mode with stirring for another 3 days in the dark. Finally, the ring ferrimagnetic vortex nanoring Fe_3_O_4_@HA nanoparticles (FVNH NPs) was separated from the waste solution using a magnet and dried using a freeze-dryer. For loading the DOX, the 5 mg DOX was added into the nanoring Fe_3_O_4_ solution in the PEI coating step and with the same following reactions.

### Characterization of FVNH NPs

The synthesized FVNH NPs were fully characterized. The microtopography was observed by scanning electron microscopy (SEM, JSM−5600LV) and electronic transmission microscopy (TEM, JEOL-2100). The crystal structure of obtained samples was determined from x-ray powder diffraction (XRD, D8 Advance Bruker) data. The dynamic light scattering (DLS, Malvern Nano-ZS90) instruments were used to measure the zeta potential and average particle size. The magnetic properties of all samples were measured on a SQUID magnetometer (Quantum Design, MPMS-XL7). An infrared thermal (IR) camera was used to record the thermal images and temperatures (Testo-875). The Nanodrop 2000 spectrophotometer was used to measure the UV-vis spectrum (Thermo Scientific). The cells were evaluated by ELISA plate reader (DNM-9606, PuLang New Technology Ltd) at 490 nm and laser confocal microscopy (FV1000, OLYMPUS).

### Magnetic hyperthermia assessed

The alternating magnetic field (AMF) was generated by using an inductive heating device (ASPG-10A-II, Shuangping). Different concentration (0–100 μg/ml) of FVNH dissolved in the water was put in the center of the coil of the AMF. After charging with fixed currents and frequency (273 kHz, 600 G), the temperatures of different samples were recorded using an IR camera. What’s more, the heating stability and the efficiency of FVNH (100 μg/ml) were also accessed with several circles of on-off of AMF (273 kHz, 600 G) and the specific absorption rates (SAR) under different field strengths (200–800 G) were calculated according to a previously reported method (Quinto et al., 2015). The SAR was calculated using following formula:

$$SAR=Cm\frac{∆T}{∆T}\times \frac{m_{v}}{m_{np}}$$

where Cm is the medium heat capacity, ΔT/Δt is the rate of temperature increase in 60 s, $m_{v}$ and $m_{np}$ are the mass of the suspension and the iron content.

### T2 relaxation and magnetic resonance imaging

In this experiment, a clinical 3.0 T MRI scanner was used for magnetic resonance experiments. To measure the T2 relaxation time, different concentrations of FVNH samples were dissolved in the water with different concertation (0.1–2 mg/ml) and then fixed with 0.5% agarose gel in 2 ml centrifuge tubes. In order to accurately measure the R2, the amount of Fe in the PBS solution before relaxation measurement was quantified by ICP-MS. T2 relaxation time was measured using Carr–Purcell–Meiboom–Gill (CPMG) pulse sequence with the following parameters: repetition time (TR) = 10,000 ms, echo time (TE) = 20, 40, 60, 80, 100, 120, 160, 200, 240 ms and the relaxation rate was obtained by fitting data points to calculate slope. For T2-weighted scan, following parameters were adopted: TR = 5,000 ms, TE = 60 ms, slice thickness = 5 mm, field of view = 50 × 50 mm, matrix = 128 × 128.

### Evaluation of the doxorubicin loading and doxorubicin releasing

To take it one step further, the obtained FVNH was explored as the drug-carrying to deliver DOX due to the large hole in the center of the nanoring. The absorption value directly measured the content of the DOX at 490 nm by a UV–Vis spectrophotometry. As previously reported (Zhang et al., 2012), the standard curve of DOX content was fitted using least squares with several known concentrations (1–200 mg/L) of DOX and corresponding absorption values at 490 nm. As mentioned above, the loading nanocarriers were collected with a magnet, and the free DOX in the supernate was separated from the nanocarriers. The AMF (273 kHz, 600 G) as an external stimulus was applied for 10 min to explore whether it would enhance the DOX release. The concentration of DOX was determined by measuring the light absorption intensity of the upper layer liquid, and the drug loading efficiency of DOX was further determined according to Eq. 1

$$DOX\;loading\;efficiency\;\left(\%\right)=\frac{Amount\;of\;encapsulated\;DOX}{Amount\;of\;FVNH}\times 100\%$$ (1)

And the cumulative release was calculated according to Eq. 2

$$cumulative\;release\;\left(\%\right)=\frac{5\overset{N}{\underset{1}{\sum }}C_{i}+50C_{N}}{M_{DOX}}\times 100\%$$ (2)

Where *N* is the number of samplings, *C* is the DOX concentration and M is the total content of DOX. The volume for the whole system and each sampling is 50 and 5 ml, separately. Three parallel tests obtain all the measurements.

### Cytotoxicity experiments

*In vitro* cytotoxicity tests of free DOX, FVNH, and FVNH-DOX were performed using 4T1 *breast* cancer cells with the standard MTT method as follows: 1×10^4^ 4T1 cells, which were in the logarithmic phase, were seeded to 96-wells plate and then different samples were added into the wells with various concentration (0–50 μg/ml). After 4 h of incubation, the previous medium was replaced by a fresh medium, and the cells were cultured for another 24/48 h. Then, 10 μl, 5 mg/ml MTT in the PBS was added to each well and incubated with the cells for extra 3 h at 37°C. The medium was abandoned, and 200 μl DMSO was added to each well to dissolve the cells. After shocking gently for 10 min, the 96-well plate was put into a microplate spectrophotometer (PULANG, DNM-9606, Beijing, China), and the absorbance values at 490 nm were recorded. Cell viability was further assessed by different treatments 1) DOX; 2) FVNH-DOX; 3) FVNH + AMF; 4) FVNH-DOX + AMF, and each group has three repetitions, and the mean value was used for analyzing. Calcein-AM/PI staining inspections were applied, to directly observe the therapeutic efficiency. Briefly, 4T1 cells were seeded to a 24-well plate at pH = 6.0 for 4 h, and then different treatments were performed as mentioned above. Subsequently, the calcein-AM and PI solution was added to each well for the double-staining, and then the dyes were incubated with cells in an incubator for another 20 min before washing off with PBS. Then the CLSM was used to observe and record the live/dead cells in the plate.

### *In vivo* experiments

The 4T1 mouse breast cancer cells (1×10^7^ per mouse) were injected into the right hind limb of 30, 4-week-old BALB/c mice. When the tumor volume reached 100 mm^3^, the mice were divided into five groups. The mice in the experimental group were intratumorally injected with 100 μl FVNH + DOX 1 mg/ml in PBS suspension and then placed in an AMF, for 10 min. For the other four control groups: 1) injected with the same volume of PBS; 2) injected with the same volume of FVNH-DOX; 3) injected with 100 μl free DOX, 4) injected with 100 μl FVNH + AMF, 5) injected with 100 μl FVNH-DOX + AMF (the amount of DOX was 5 mg/kg, AMF: 273 kHz, 600 G, 10 min). The treatment was repeated the day after the first treatment to enhance the effect. Five major organs and tumor tissue were excised for H&E staining. Twenty days later, all tumor-bearing mice were sacrificed. The length (L) and width (W) of the tumors were recorded every 2 days using vernier calipers, as well as the weight of the mice was monitored. The tumor volume (V) was estimated by the following formula: V= (L×W^2^)/2. The relative volume of the tumor was calculated as V/V_0_ (V_0_ was the initial tumor volume before treatments for each mouse).

### Statistical analysis

The measurement values were presented as mean ± standard deviation (SD). All statistical analysis was performed by using *t-test* analysis. *p < 0.05 and **p < 0.01 were accepted as significant and high significant level.

## Results and discussion

### Synthesis and characterization of FVNH

**FIGURE 1:**
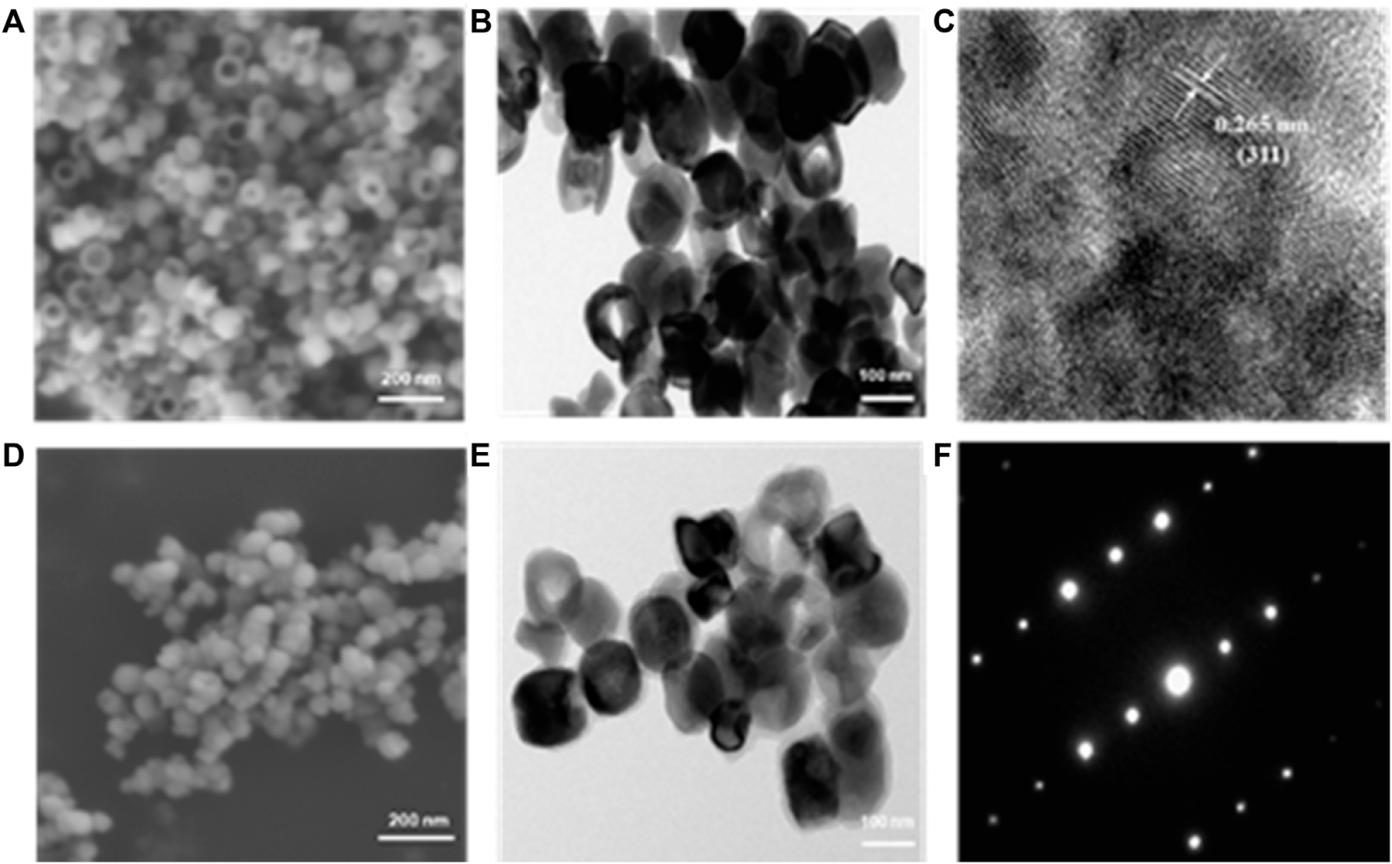
Morphology images of obtained samples. SEM **(A)** and TEM **(B)** images of naked nanoring Fe_3_O_4_. **(C)** High-resolution TEM image recorded on Fe and the interlayer spacings matched with that of magnetite. SEM **(D)** and TEM **(E)** images of HA-coated nanoring Fe_3_O_4_ and **(F)** corresponding fast Fourier transforms electron diffraction patterns.

The ring shape of nanoparticles forms progressively as expected through a classical nucleation-aggregation-dissolution process under high pressure and temperature condition. Specifically, the nano hematite disks were first formed by the FeCl_3_. The electronegative ions, SO₄^2^⁻ and PO₄³⁻, will coordinate competitively with the surface iron and replace the oxygen. Finally, as previously reported, the ring hole is formatted after the reaction duration can control dissolution accrued on the exposed crystal plane and the hole size (Jia et al., 2008). The morphology of ferrimagnetic nanoring is observed by SEM (Figure 1A), and the uniform ring shape of nanoparticles can be clearly seen. The average external and inner diameters are around 119 ± 11 nm and 69 ± 7 nm. The ring thickness and height are about 32 ± 4 nm and 82 ± 6 nm. The TEM image and high-resolution TEM image of the ferrimagnetic nanoring are shown in Figures 1A,B. Besides the ring shape of the synthesized nanoparticles being further confirmed, the d-spacing of lattice fringes is 0.265 nm (Figure 1C), which is in line with (311) plane of Fe_3_O_4_. After coating with the HA, no significant changes were observed in SEM images (Figure 1D). However, a layer of a mistlike slice, about 11 nm thick, can be identified on the TEM image (Figure 1E). The fast Fourier transforms electron diffraction patterns (Figure 1F) display well-defined sharp diffraction spots and thus further verifying that the synthesized nanoring Fe_3_O_4_ particles are single crystals.

**FIGURE 2:**
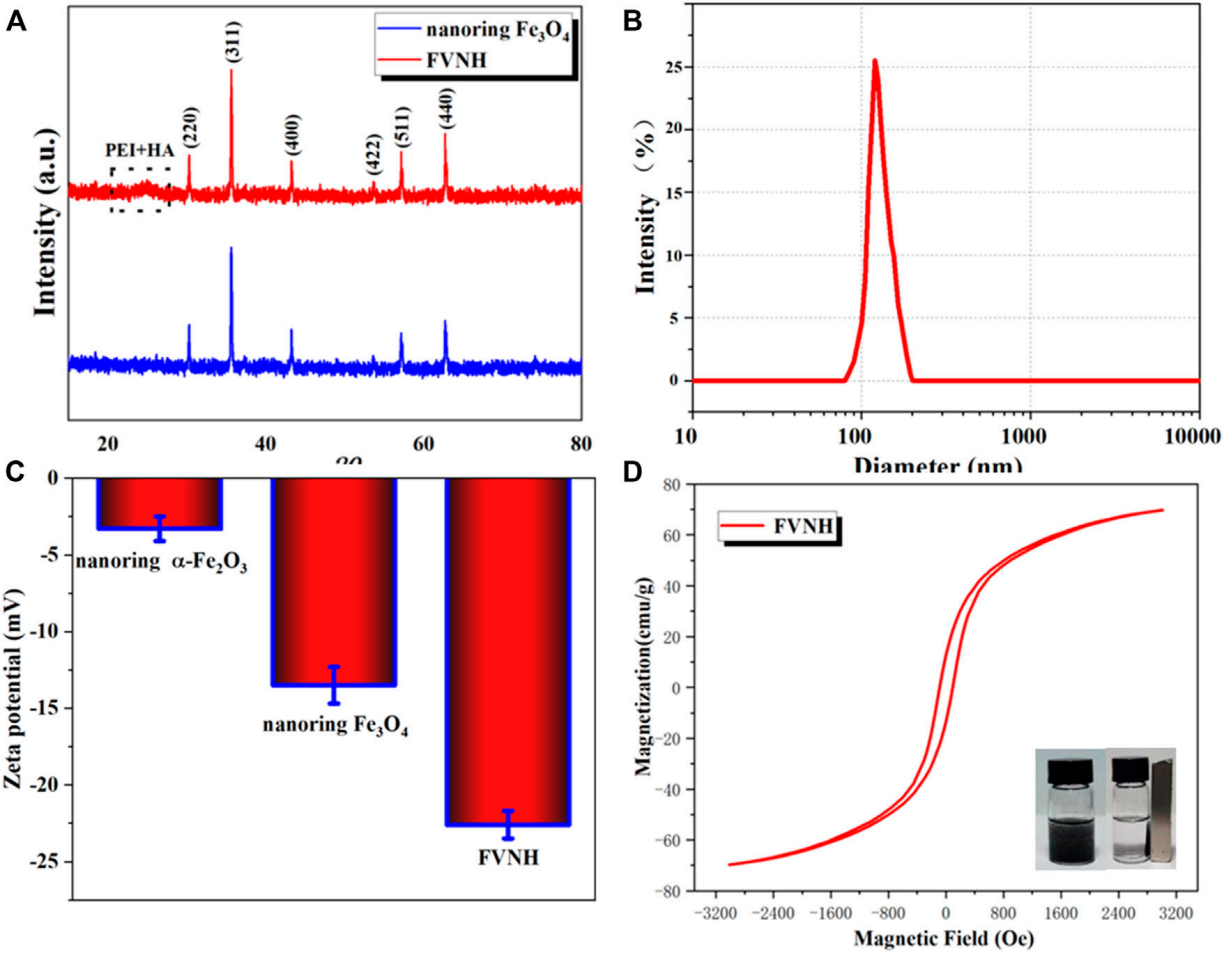
**(A)** X-ray diffraction patterns of naked nanoring Fe_3_O_4_ and FVNH. **(B)**The hydrodynamic diameter distribution of synthesized FVNH in water measured by DLS. **(C)** Zeta potentials of the nanoparticles for different synthetic steps. **(D)** The hysteresis curves of Ferrimagnetic FVNH nanoparticles and photographs of the FVNH solution in PBS with and without a magnet nearby.

The XRD technique was used further to analyze the nanoring crystal structure and its derivatives. As shown in Figure 2A, all the diffraction peaks belong to the crystal peaks of Fe_3_O_4_. It should be noted that there were no other peaks, which further showed that the compositions of both samples are pure. For the FVNH, the XRD pattern is very similar to the naked nanoring Fe_3_O_4_, except for a slightly elevated envelope between the 20–30-degree region caused by the PEI and HA, which was caused by the PEI and HA together. Since the surface charge is one factor influencing the materials entering cells, the zeta potential of different samples was measured. What’s more, consistent with previous studies (Jiang et al., 2008; Yeh et al., 2013), HA was successfully decorated to the nanoring Fe_3_O_4_-PEI, and the zeta potential dropped down to about –13.5 mV from 18.2 mV accordingly. After the FVNH was dispersed in water, the average hydrodynamic diameter was 181.2 nm measured by DLS (Figure 2B), much higher than the size measured from the SEM image. This discrepancy can be attributed to the invisible shell layer for the latter. What’s more, the colloidal stability of FVNH was explored by monitoring the hydrodynamic diameter over 1 week and the result demonstrate the suitable stability (Supplementary Figure S1). As shown in Figure 2C, the zeta potential for naked nanoring α-Fe_2_O_3_ and Fe_3_O_4_ is −3.3 mV and −13.5 mV. After the polymerization of PEI and HA, the zeta potential decreased to −22.6 mV. Theoretically, the negative charge of FVNH will aid the NPs enter cells (Asati et al., 2010; Prijic et al., 2010). As shown in Figure 2D, the hysteresis loops of FVNH were evaluated. FVNH show relatively large saturation magnetization, 69.8 emu/g, which means that the FVNH may have great potential to enhance the MHT and MRI. What’s more, both coercivity and remanence of FVNH are very low. Thus stable magnetic sol–gel can be realized by reducing the dipole-dipole interaction like the superparamagnetic iron oxide nanoparticles. In addition, as shown in the photo, FVNH can be easily absorbed by a magnet, further indicating that the synthesized material has excellent magnetic properties, which can be used for tumor magnetic target treatment.

**FIGURE 3:**
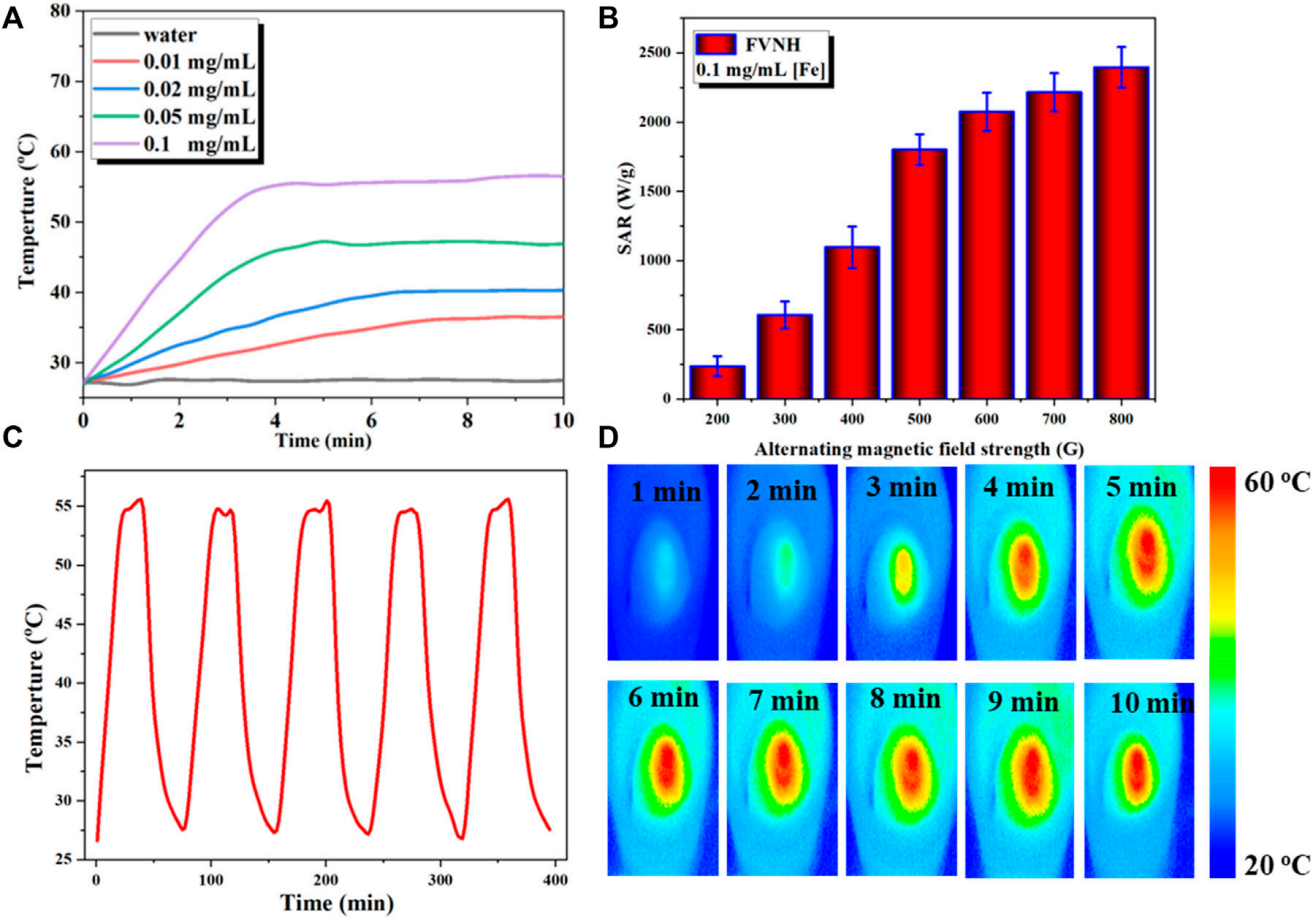
**(A)** Temperature increase profiles for water and different concentration of FVNH under AMF (273 kHz, 600 G) for 10 min. **(B)** Specific absorption rate dependence on the amplitude of AMF. **(C)** Magnetothermal stability of FVNH over a repeated magnetic field on/off cycles. **(D)** Photothermal images of FVNH suspension (200 μg/ml) under AMF (273 kHz, 600 G) for different time durations.

Subsequently, the magnetic-thermal property of FVNH under the AMF was explored, and the results are shown in Figure 3. The temperature rose rapidly in the initial several minutes and then reached to flattened phase gradually within 10 min. Figure 3A shows the temperature-increasing profiles of AMF-caused heat generation for different FVNH concentrations (0–100 μg/ml). Furthermore, the temperature changes were recorded with fixed strength of 600 G, as shown in Figure 3A, the faster rate of temperature increases with the increase of FVNH concentration. For the same FVNH concentration (100 μg/ml), the higher magnetic field strength will increase a magnetic heating effect, and higher SAR values can be achieved (Figure 3B). The high SAR values of FVNH can be attributed to two factors: 1) the magnetic vortex structure generates large hysteresis loss, and 2) the magnetization reversal process of vortex-to-onion. The magnetothermal stability of FVNH dispersion was also investigated, as can be seen in Figure 3C, the temperature of FVNH dispersion raised from room temperature (∼26°C) to the maximum balance temperature (∼55°C) during the five cycles of AMF on-off. It can be preliminarily determined that the proposed FNVH has high magnetothermal stability, which may be suitable for multiple treatments with only one injection. What’s more, the thermal images of different times (100 μg/ml, 600 G) are shown in Figure 3D. When the AMF duration was increased from 0 to 10 min, the thermal images became much hotter, which is in line with the temperature-increasing curves. Preliminarily, the obtained FVNH shows excellent magnetic heating characteristics and may have potential application in MHT.

### T2 weighted magnetic resonance images

**FIGURE 4:**
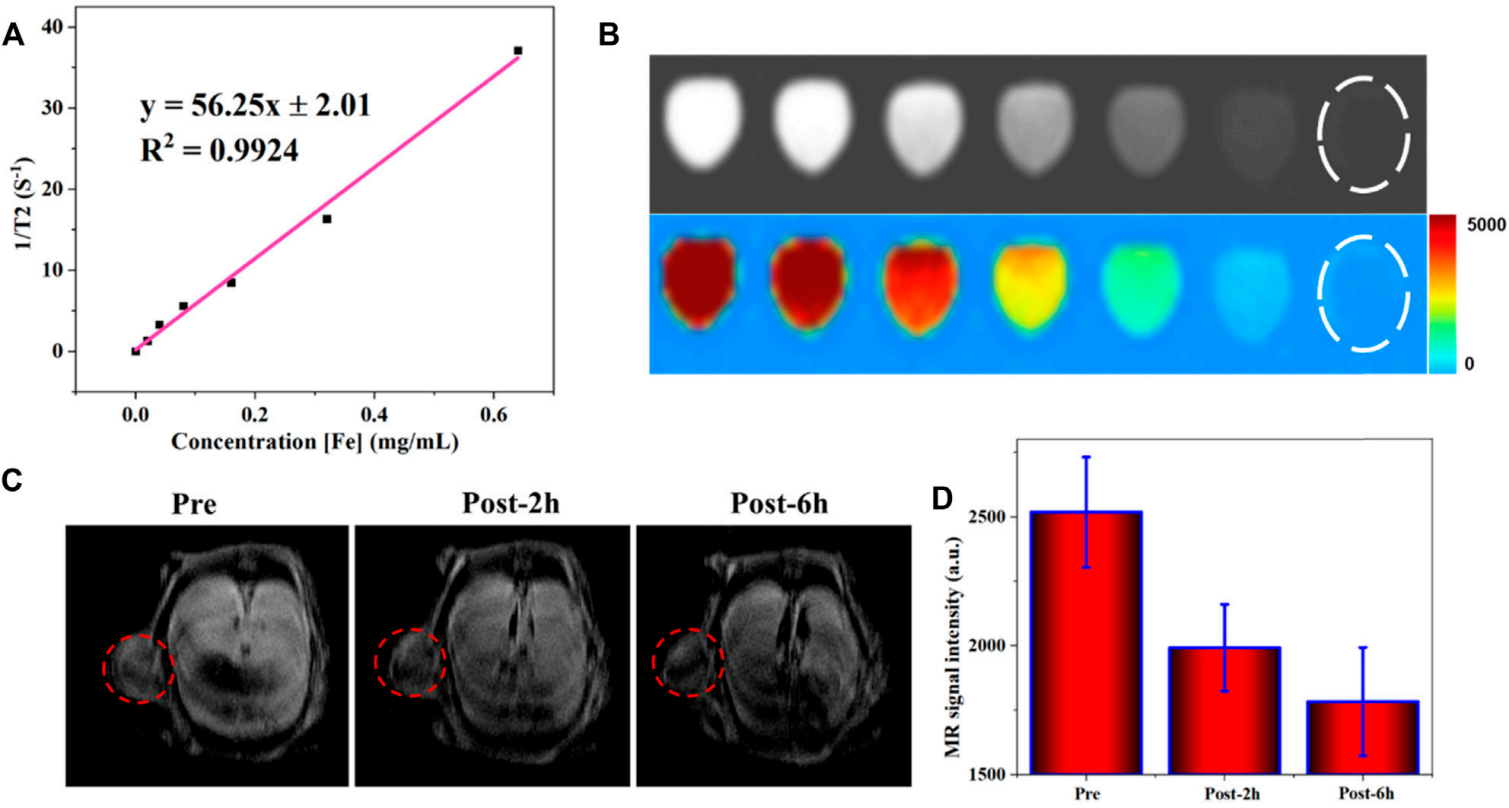
MR contrast enhancing ability of FVNH. **(A)** The transverse relaxation rate (1/T2, s-1) as a function of Fe concentration (mM) for FVNH at clinal 3.0 T MRI scanner. **(B)** T2W MR and pseudo-color images at Fe concentrations. **(C)** *In vivo* MR (T2W) images in cancer before and after injection of FVNH NPs. **(D)** MRI signal intensity changes in the tumor region.

For modern medicine, MRI plays an essential role in tumor diagnosis. Besides the morphological examination, MRI is the primary way to evaluate benign and malignant conditions through the injection of the contrast agent. Various gadolinium-based small molecules are currently used as T1W contrast agents, which will make the tissues much brighter but have biological security problems. On the other hand, the T2W contrast agent developed slowly. Superparamagnetic nanoparticles, well known for their good biocompatibility, are considered an ideal contrast agent that can efficiently shortenthe T2. As a similar magnetic material, the potential of obtained FVNH as T2 contrast agents were preliminary explored in this work at 3 T. As shown in Figure 4A, the T2W images become dark gradually as the concentration of the FVNH increase. Additionally, the transverse relaxivity (r2) value of the FVNH NPs reached 56.25 mM^−1^ S^−1^, which indicats that the synthesized material has a great ability to shorten T2. The amount of relaxation change depended on the distance between the Fe of nanoring Fe_3_O_4_ core and the nearby proton of water. Due to shielding effects from the PEI-HA shell, the saturation magnetization decreased slightly, and the transverse relaxation rate decreased accordingly for the same reason, which is consistent with previous studies. It should notice that the FVNH also shows higher T2 values than conventional spherical iron oxide nanoparticles, which can be attributed to the vortex property. T2W images are shown in Figure 4B, with the increase of FVNH concentration from left to right tubes, the image becomes darker. Then the sample was injected into a tumor-bearing mouse, as can be seen in Figure 4C, after the injection, the tumor (red dash circles) showed much lower MRI signal intensity, which means FVNM NPs have been concentrated in the tumor site. Additionally, MR signal intensity is significantly lower after 6 h injection than that of 2 h injection (Figure 4D), which indicates the sample concentration is higher for the former time point. The results revealed that FVNH NPs had an outstanding performance for enhancing T2W MRI both *in vivo* and *in vitro*, and could serve as a potential contrast agent for MR image-guided MHT.

### Drug loading and release

**FIGURE 5:**
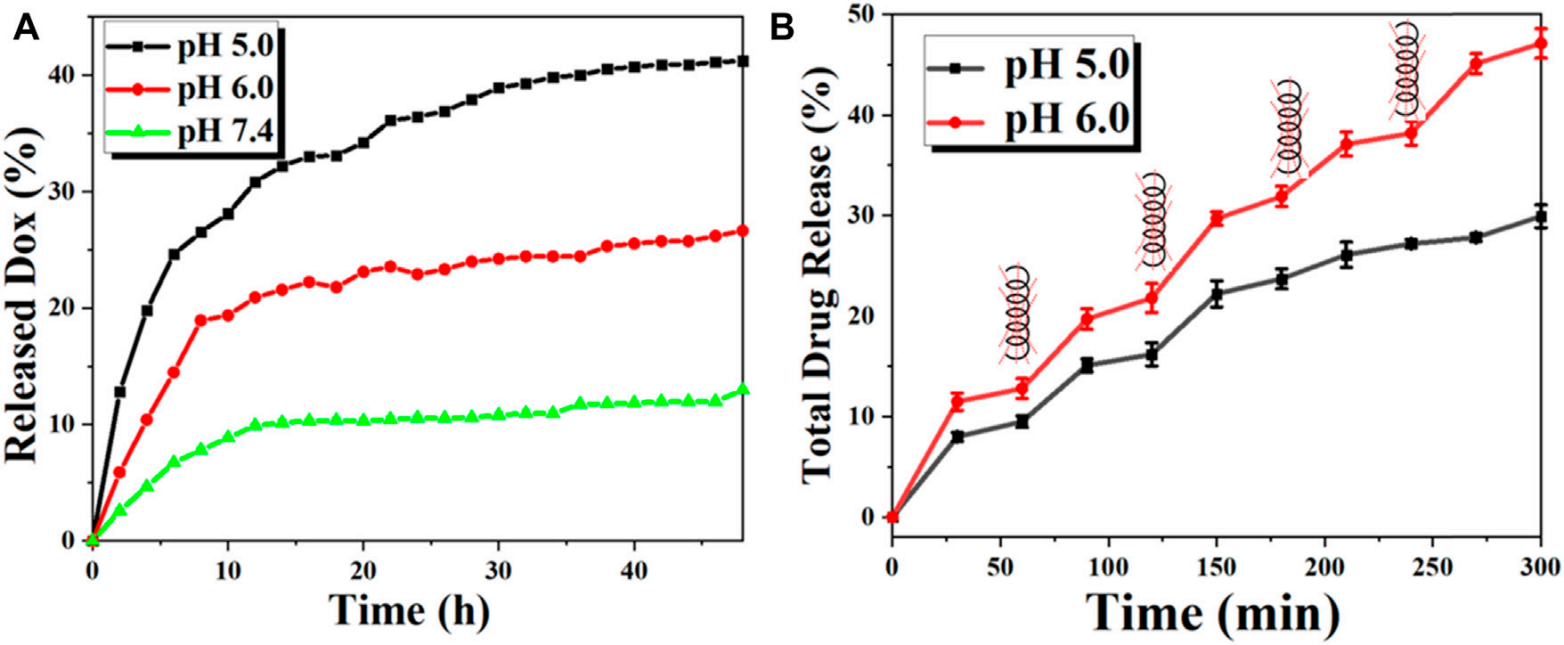
The *in vitro* DOX release behavior of the FVNH-DOX. **(A)** Cumulative DOX release profiles of FVNH-DOX at different pH within 2 days and **(B)** In the presence and absence of AMF with a 400 kHz 600 G at different time points (each time point lasts 10 min) under different pH within 5 h.

The UV-Vis spectrophotometer evaluated the DOX loading in FVNH by the absorption value at 490 nm. Moreover, the calculated loading capacity of DOX for the synthesized NPs is 31.5%. The relatively high value of the drug loading capacity can be attributed to two factors: 1) There is a very large hole in the center of the ring shape basic nanomaterial; and 2) The other may be the intermolecular interactions, like the hydrogen bonding interaction. The *in vitro* DOX release behaviors were explored at different pH (7.4, 6.0, and 5.0) using dialysis bags. As shown in Figure 5A, the release profiles were plotted for 48 h duration according to the absorbance values. The fluorescent intensity was much higher for pH 5.0 than 6.0 and 7.4, which means the lower value environments will accelerate the DOX release. The cumulative released amount in 48 h is 13.0%, 26.6% and 41.2% for pH 5.0, 6.0, and 7.4, separately. It should be noticed that the efficient release under acidic conditions is very adaptable to the tumor microenvironments. The results are in line with previous similar studies (Fang et al., 2019; Wu et al., 2019). This phenomenon is attributed to the interaction effects between DOX and nanocarrier disappearing and the dissociation of carboxylic acid groups. The responses of drug release excited by the magnetothermal thermal were assessed subsequently. As shown in Figure 5B, the fluorescence intensity shows a leaping increase when applying AMF, while the leaping phenomenon disappears when the AMF is absent. The smart “on-off” effect may be that the increased temperature will debilitate the interaction between DOX and nanocarrier, weakening the electrostatic force.

### Cell viability assay

**FIGURE 6:**
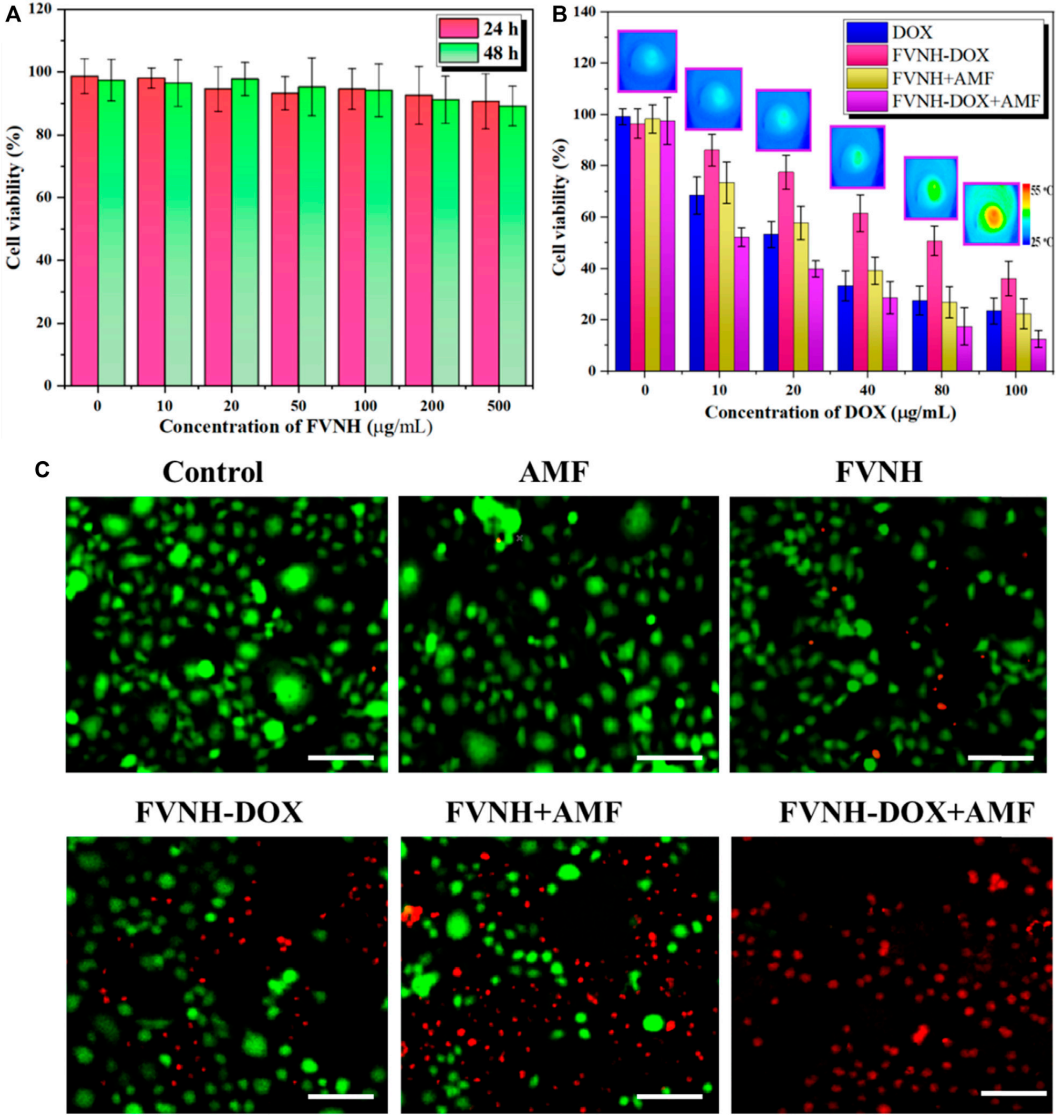
Cell viability assay. **(A)** Cell viability after incubation with different FVNH for 24 and 48 h. **(B)** Cell viability upon various kinds of treatments at different concentrations of DOX and representive thermal images of FVNH-DOX + AMF group. **(C)** Fluorescence images of co-stained with calcein-AM (green) and PI (red) 4T1 cells after different therapies. (*n* = 3, mean ± SD), (Scale bar = 100 μm).

Subsequently, we studied the cell viability with various treatments by the standard MTT method, and the results are shown in Figures 6A,B. FVNH has a negligible effect on the 4T1 cell viability even at a concentration of 500 μg/ml and 48 h incubation, which preliminary evidence it is excellent biocompatible nature. The amount of iron internalized in the cells was quantified using ICP-MS. As shown in Supplementary Figure S2, higher cellular iron levels were observed with higher FVNH concentration from 0 to 0.2 mM [Fe] as expected. While for different treatments (free DOX, FVNH-DOX, FVNH + AMF, FVNH-DOX + AMF), with the decrease in the concentrations of samples (conversion of DOX content), the cytotoxicity for 4T1 cells increased accordingly. As expected, for FVNH-DOX + AMF group, the cell viability is significantly lower than in any other group. That is to say, the synergistic therapy is better than each independent treatment. In order to observe the cell killing, a live-dead cell stain was further applied as well. As shown in Figure 6C, the live cells were stained with green color, and the dead cells were stained with red color. The largest red fluorescence area appeared in the synergistic group, which is consistent with the MTT experiments. Altogether, these results proved that the designed FVNH-DOX had a very good combined MHT-chemo effect for *in vitro* antitumor cell efficiency.

### The *in vivo* therapeutic efficacy 

**FIGURE 7:**
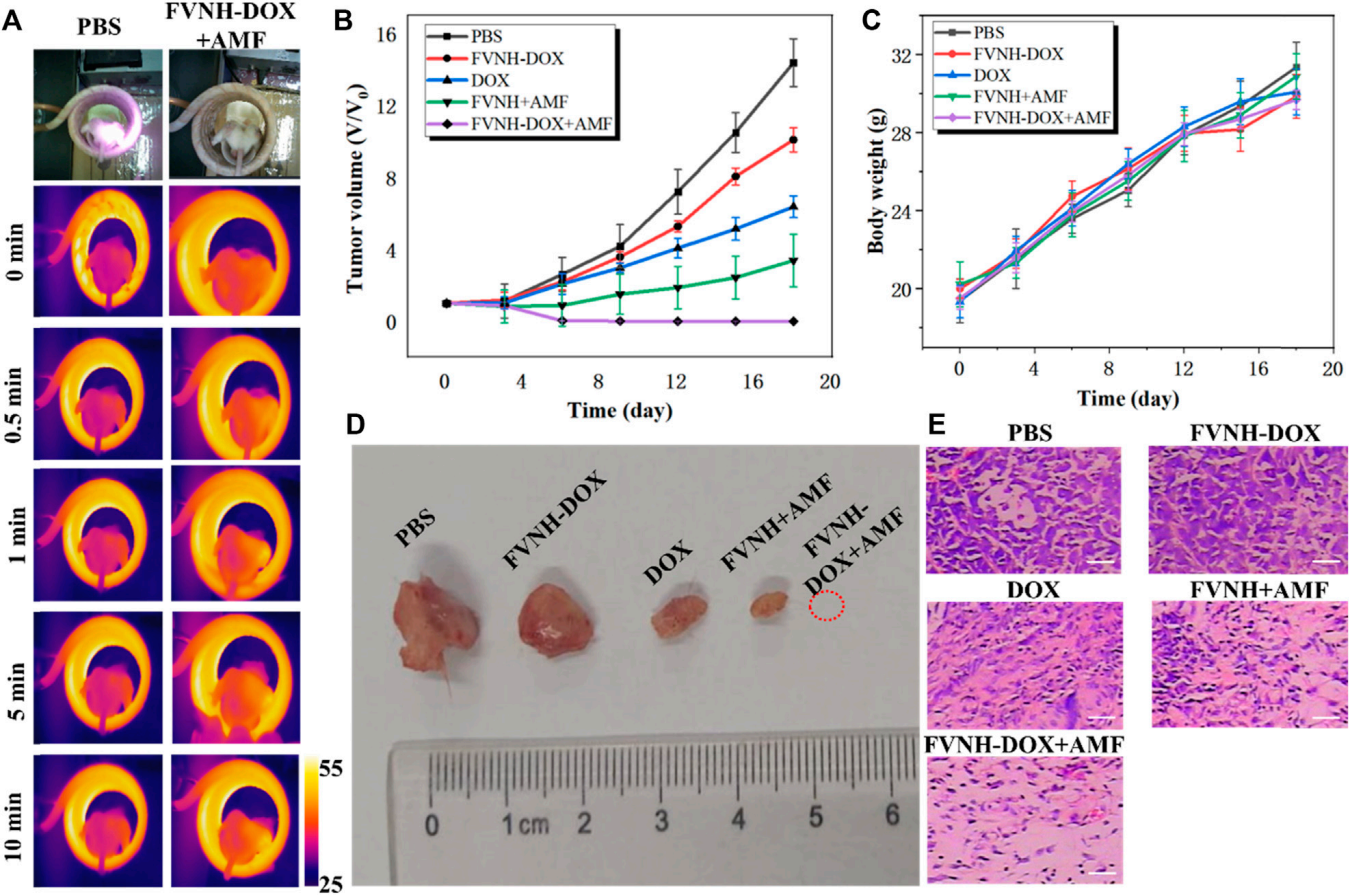
*In vivo* cancer magnetothermal-chemotherapy in tumor-bearing mice models with 4T1 cancer cells. **(A)** Photos and corresponding thermal images of tumor-bearing mice under AMF at different time points after injection with PBS or FVNH-DOX; **(B)** The relative tumor growth curves of different groups; **(C)** Body weight curves for different groups after treatment; **(D)** Representative tumors picture from different treatment groups after the experiments; **(E)** The H&E images of tumors collected from different treated groups. (Scale bar = 100 μm).

*In vivo* combined antitumor tests were performed on tumor-bearing mice using FVNH-DOX as a therapeutic reagent. To monitor the magnetothermal effect of synthesized FVNH *in vivo*, temperature changes in the tumor site under the AMF were recorded using an IR camera. As shown in Figure 7A, the temperature of the tumor increased rapidly under the AMF, rising significantly from approximately 37°C to about 45°C within 1 min. In contrast, the temperature of the control tumors remained almost unchanged under the same AMF. As tumor cells are more heat sensitive than normal cells, these results further demonstrate the promise of FVNH-DOX as an effective agent for both chemo-magnetothermal therapy. Although the magnetic nanorings have been used as magnetothermal agents to treat tumors, the potential use of it encapsulated with DOX for synergistic cancer treatment has not been reported. The changes in relative tumor volumes in 4T1 tumor-bearing mice further demonstrated the anti-tumor capacity (Figure 7B). All treatment groups showed a slight overall increase in body weight over the 18-day study (Figure 7C), further indicating few side effects of the FVNH and the magnetothermal treatment. The FVNH-DOX + AMF treatment group showed significant tumor suppression after 18 days of treatment, but for the other four groups, PBS, FVNH-DOX, free DOX, and FVNH + AMF groups still had various degrees of tumors growth. The FVNH-DOX + AMF treatment group had the smallest tumor volume, even disappeared (Figure 7D), of the five groups, confirming that the combination treatment was much better than the other single treatment groups. Furthermore, as seen in the H&E-stained tumor tissue sections, there was more necrosis and apoptosis in the tumor tissue of the FVNH-DOX + AMF treated group (Figure 7E), but no significant necrotic cells in other major organs (Supplementary Figure S3). Histological analysis of diferent organs in tumor-bearing mice stained with H&E are displayed in Supplementary Figure S3. All the organs from five gruops were normal, preliminary indicating that less toxicity of FVNH and treaments to the major organs. Overall, the FVNH loading with DOX nanocomposites in the present of AMF could be a promising nanoplatform for combined magnetothermal-chemotherapy of cancer in future clinical trials.

Due to the lack of depth limitation, low cost, and suitability for remote control, magnetothermal therapy, and chemotherapy, based on magnetic nanoparticles in combination with controlled drug delivery systems, have been extensively studied in recent years. To improve the biocompatibility of magnetic nanoparticles and to achieve superior diagnostic and therapeutic purposes, scientists have made a number of material-based strategies. Fan et al. (2010) and our previous study demonstrated that the ring shape nano magnetite has magnetic vortex properties and thus excellent magnetothermal properties for magnetothermal tumors therapy as well as enhancing MRI (Bao et al., 2021); By modifying HA on graphene and attaching the composite to iron oxide nanoparticles after wrapping DOX, Pramanik et al. found that this nanoplatform could not only control the drug release by an external magnetic field, but it would kill much more breast cancer cells than the non-magnetic thermal control group (Pramanik et al., 2019). Recently, Zhang et al. (2020b) synthesized an iron oxide nanoparticle with super magneto-thermal efficiency through a green biomineralization process, with a specific absorption rate of 2390 W/g, and they demonstrated that the NPs could be used as an excellent magneto-thermal agent and nanoenzyme as well for liver tumor inhibition. Despite the great progress made in the field of nanomediated magnetothermal therapy, more time may be needed for its application in the clinic, and there are still challenges to be addressed, such as the low thermal efficiency compared to photothermal and biosafety issues.

## Conclusion

In this paper, the one-pot solvothermal method combined with the following reduction reaction was used to prepare ring shape and mono-dispersion Fe_3_O_4_ nanoparticles. The core-shell nanoring Fe_3_O_4_@HA loaded with DOX was formed for magnetothermal-chemo cancer therapy and enhancing T2W MRI imaging. Notably, the FVNH shows significantly extremally high magnetothermal conversion efficiency and shoring the transverse relaxation time. Additionally, a thin HA coating was formed on the nanoring surface, which endowed the NPs with a high-capacity of drug loading and triggered releasing abilities. The preliminary results demonstrated that the established vortex nanoring Fe_3_O_4_-based nanoconstruct might hold great potential for DOX delivery and cancer theranostics.

**SCHEME 1:**
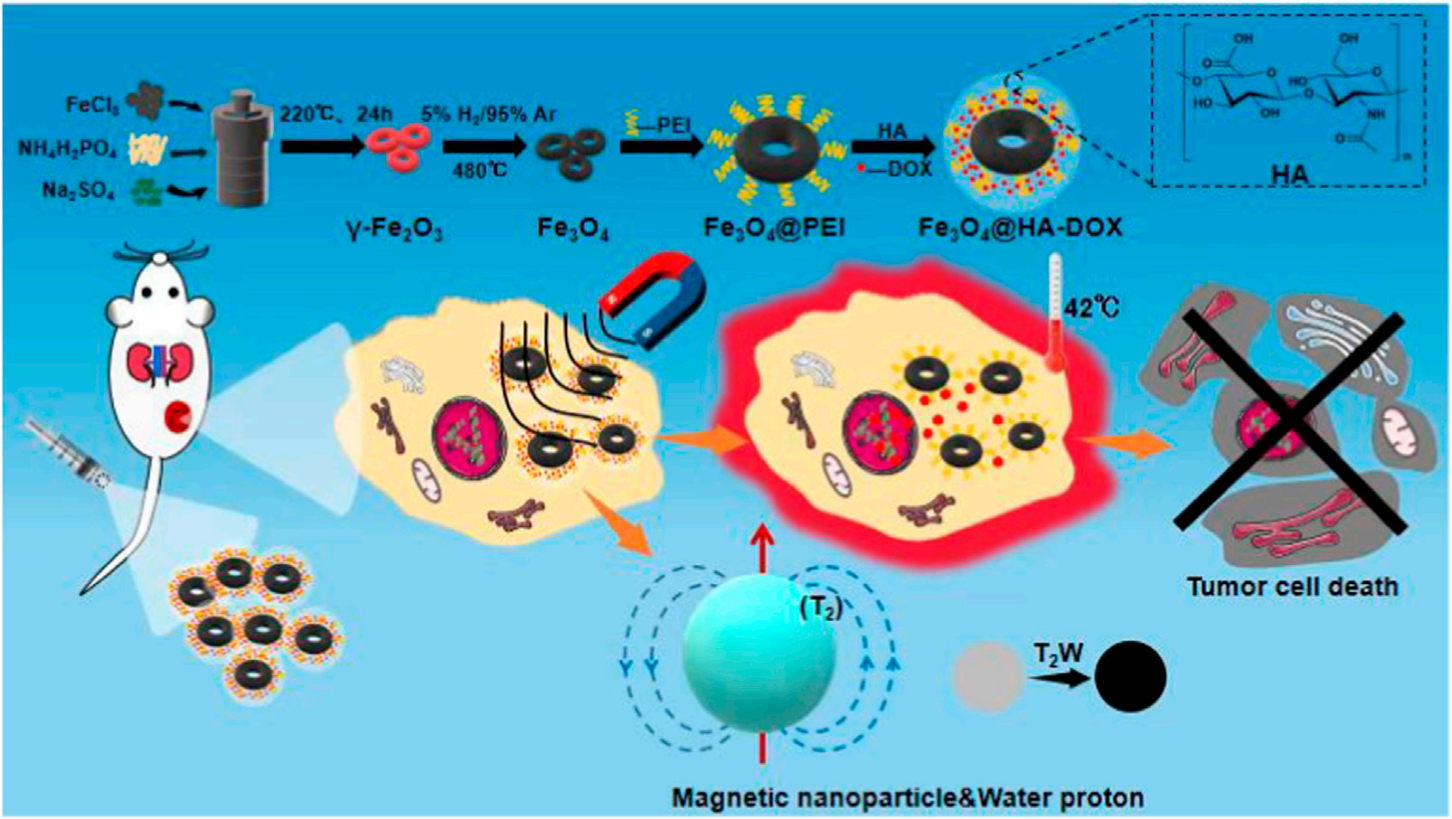
Schematic illustration of the nanoring Fe_3_O_4_@HA-DOX for MRI imaging-guided synergistic MHT-chemo cancer therapy.

## Data Availability

The original contributions presented in the study are included in the article/Supplementary Material, further inquiries can be directed to the corresponding authors.
