# Supplementary material for: Interfacial engineered iron oxide nanoring for T2-weighted magnetic resonance imaging-guided magnetothermal-chemotherapy
Source: Front Bioeng Biotechnol. 2022 Oct 6;10:1005719. doi: 10.3389/fbioe.2022.1005719 (PMC9582775; doi:10.3389/fbioe.2022.1005719)

**Supporting Information**

**Interfacial engineered iron oxide nanoring for T2-weighted magnetic resonance imaging-guided magnetothermal-chemotherapy**

Table of Contents:

| 1. Variation of FVNH hydrodynamic diameter with time. | S1 |
| --- | --- |
| 1. ICP-MS analysis of the amount of intracellular iron content. | S2 |
| 1. Histological analysis of five major organs. | S3 |

1. Figure S1. Variation of FVNH hydrodynamic diameter with time.


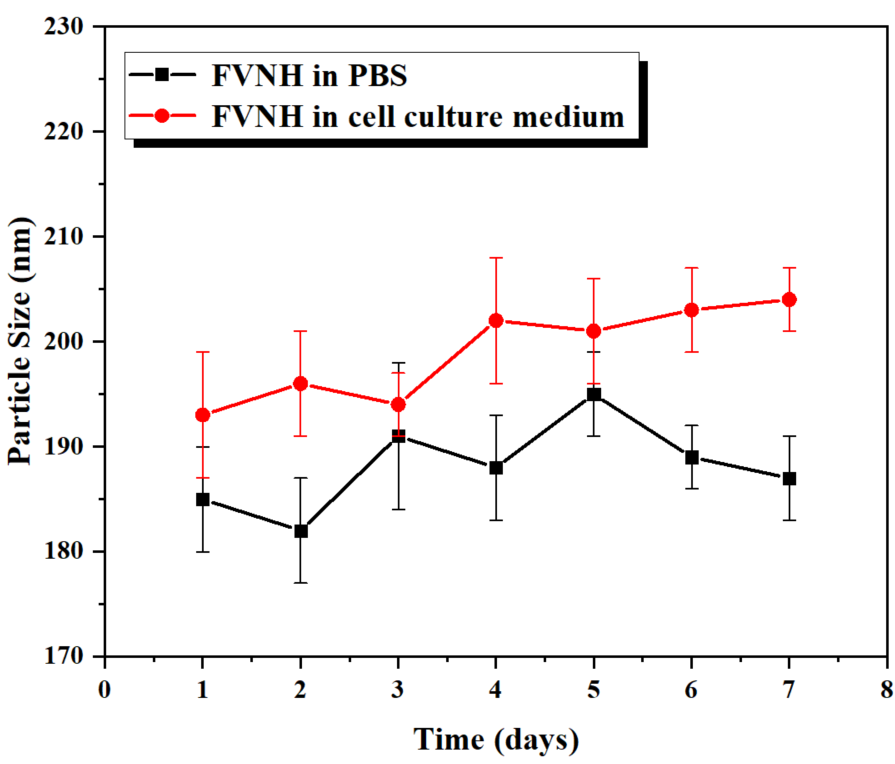


1. Figure S2. ICP-MS analysis of the amount of iron in the cells.


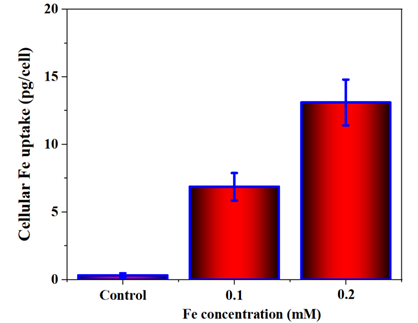


1. Figure S3. Histopathological analysis of the main organs in different groups. (Scale bar = 100 µm)


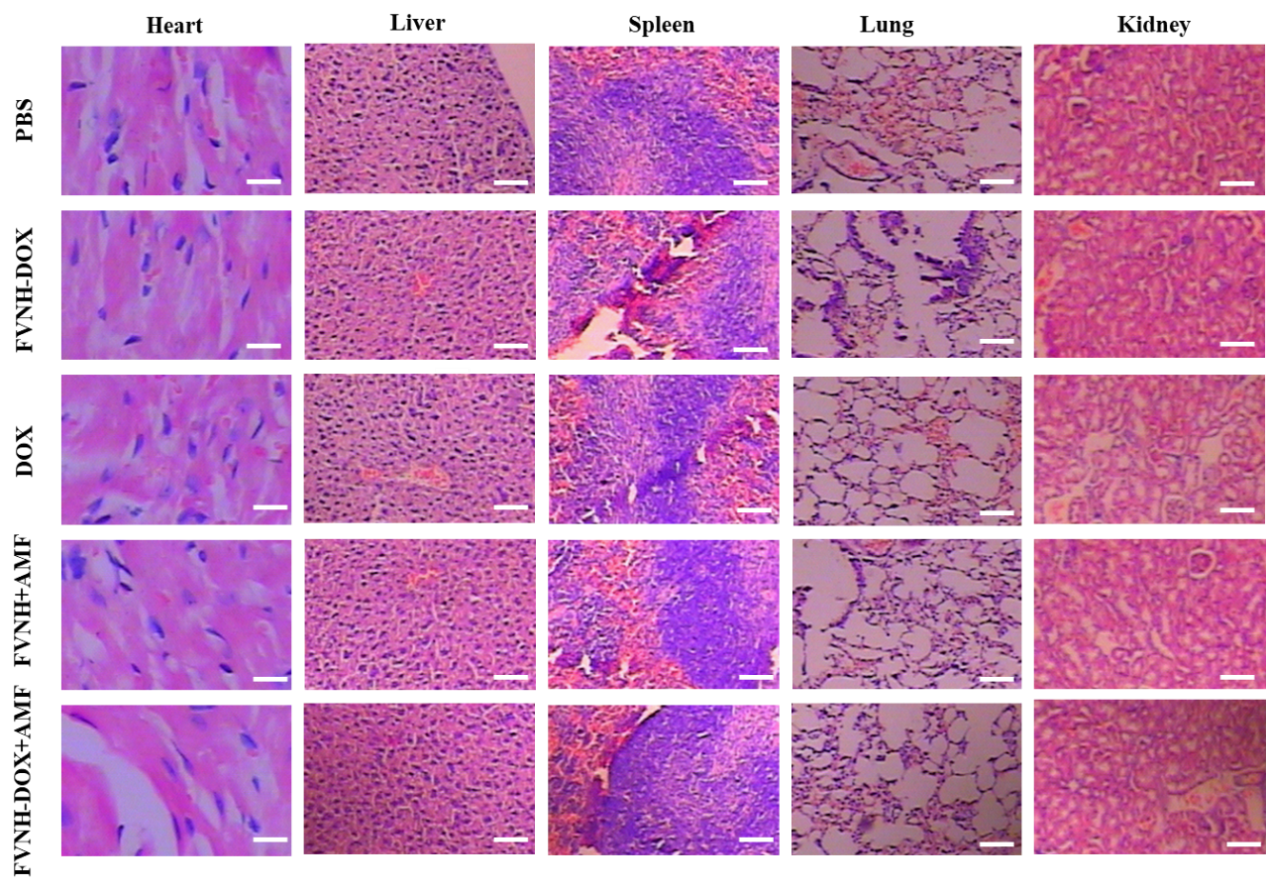

Supplement: Supplementary file 1 [file DataSheet1.docx]
